# Oral Care of Hospitalised Older Patients in the Acute Medical Setting

**DOI:** 10.1155/2013/827670

**Published:** 2013-05-30

**Authors:** Kathryn Salamone, Elaine Yacoub, Anne-Marie Mahoney, Karen-leigh Edward

**Affiliations:** ^1^Austin Health, P.O. Box 5555, Heidelberg, 3084 VIC, Australia; ^2^Clinical Education Unit, Austin Health, P.O. Box 5555, Heidelberg, 3084 VIC, Australia; ^3^Nursing Research Unit, Faculty of Health Sciences, Street Vincent's Private Hospital, Australian Catholic University, VECCI Building, Locked Bag 4115, Fitzroy MDC, Melbourne, 3065 VIC, Australia

## Abstract

Oral health care is an essential aspect of nursing care. There are many variances in the quality and frequency of the oral care that is delivered to patients by nursing staff, such as oral care being given a low priority when compared to other nursing care elements, oral care being neglected, and oral care delivery being dependent on the nurse's knowledge of oral hygiene. Additionally, there are some particular patient groups known to be at risk of oral health problems or who have existing oral diseases and conditions. As people age their susceptibility increases to chronic and life-threatening diseases, and they can be at increased risk of acute infections increases compromised by ageing immune systems. The aim of this literature review was to ignite the discussion related to the oral care practices of nurses for older acute medical hospitalised patients. The review revealed that nursing staff know that good nursing includes oral health care, but this knowledge does not always mean that oral health care is administered. Oral health care seems to be separated from other nursing activities and is not discussed when nursing care plans are written, only when oral problems are obvious.

## 1. Introduction

The aim of this review of the literature is to ignite the discussion related to the oral care practices of nurses for older acute medical hospitalised patients. This paper explores the literature related to oral health care for older patients admitted to acute medical wards. Caring for older patients with complex medical issues is challenging and one fundamental basic care is the provision of oral care which is often overlooked. Literature relevant to this issue was reviewed to confirm our view that oral care was often overlooked and could be improved.

 As people age, their susceptibility to chronic and life-threatening diseases as well as acute infections increases, exacerbated by compromised immune systems. Tooth loss and periodontal disease are also prevalent in the older population [1]. The number of elderly people in society is increasing and consequently also the number of older people in need of health care and nursing care [2]. It is projected that by 2025, the number of elderly people will increase by 146% to 1.25 billion worldwide [3]. Over the past two decades in Australia, the number of elderly people has increased by 170.6% [4]. The growth in this population of older people is staggering, posing tremendous challenges in caring for this group and their chronic conditions. 

The consequences of chronic diseases and conditions are significant, leading to disabilities and reduced quality of life. Individuals with the most prevalent medical problems tend to have the highest rates of oral disease, with an association between poor oral health and adverse medical outcomes such as aspiration pneumonia and cardiovascular disease [5, 6]. Attention has been focused on oral care as the evidence accumulates to support an association between the bacteria in the mouth and those respiratory pathogens that cause pneumonia [7]. The benefits of this literature review can bring to light practice gaps, and areas for practice improvements for nursing care of this vulnerable group, through research, quality improvement activities, and development of practice guidelines within a policy framework. 

## 2. Literature Search

A search of the literature used the search terms *oral health* AND/OR *oral hygiene* AND *nursing* AND *medical patients* published between 2006 to 2012 in the databases of CINAHL and Medline. There were over 600 articles retrieved on oral hygiene/health; however there was limited literature that specifically focused on oral care for older medical patients in acute care. The literature in this review was obtained from nursing, medical, dental journals and government publications, and grey literature discussing oral care, hygiene, and inpatients. The literature excluded from this review was articles discussing oral health for children, and oral surgery.

## 3. Good Oral Health

Good oral health is important. Having a clean and healthy mouth contributes to a sense of well-being [8–11] allows for fluid and nutritional intake, assists with communication and quality of life [9], and assists with clear speech and communication [10]. Paulsson et al. [12] note that maintenance of good oral health is important for patients in hospital, as it contributes to the well-being, recovery, and nutritional needs of the patient, and it requires the involvement of nursing staff.

The literature suggests that oral care is not a highly technical skill or requires huge resources [9]. It is an individualised and practiced behaviour [13] and is an essential aspect of nursing care [9, 14, 15]. As Dickinson et al. [16] state, when a person is unable to perform their own oral care in hospital it becomes the responsibility of nursing staff. Bissett and Preshaw [8] suggest that oral care is like other personal care needs such as bathing and toileting; it is an essential component of holistic care [14]. There are variances in the quality and frequency of oral care delivery to patients. These variances in oral care relate to different factors, such as oral care being neglected [17–19] and oral care being given a low priority when compared to other nursing care elements [7, 8]. Oral care is dependent on the nurse's knowledge of oral care best practice. Fitzpatrick [20] acknowledges that nurses' knowledge of oral hygiene is variable. Oral hygiene is often thought to be underrecognised by nurses for the fundamental impact it can have on a person's wellbeing and health status [18]. In this context, poor knowledge has the potential to compromise the quality of patient care [21].

The delivery of oral care to hospitalised patients is recognised in the nursing literature as an imperative to maintaining health and wellbeing [22, 23] particularity in vulnerable groups of patients who cannot maintain their own oral health when hospitalised. There are some particular patient groups known to be at risk of oral health problems or who have existing oral diseases and conditions. These specific patient groups are cancer patients [24], palliative patients [13], patients who are intubated [17, 25], critically ill patients, frail patients [12], and the elderly [9, 26]. 

Research evidence suggests there are subgroups of older people known to be at risk of poor oral care, in particular people with dementia [27], and those that have come from residential care [20, 28–30]. It is often these subgroups of older people that are a majority of the patients on acute medical wards. Acute medical wards in hospitals are frequently filled with frail people, and this group of patients have been noted to often have comprised oral health (Andersson 1999 and Öhrn et al. 2001 as cited in Paulsson et al. [12]). Fitzpatrick [20] noted that older adults due to ageing processes have a loss of soft tissue attachments, which results in loosening of teeth root exposure, and teeth can become more brittle. Due to the ageing process older people have oral care needs that need to be met [26]. Common side effects of poor oral care are pain, difficulty with swallowing, poor or compromised nutritional intake [31], infection [26], systemic infection [23], and impaired communication.

## 4. General Medicine: Oral Health

In the acute medical setting, nursing staff are responsible for assisting with oral health care. Admission to hospital is not only a time for the active management of the presenting disease but also an excellent opportunity for the health promotion and screening for undetected pathology. Preston et al. [32] discuss the importance of nurses performing daily oral care for older people on acute, subacute, and rehabilitation wards; however these authors recognise that much of the nurses education in this important area has been provided during their early training and regular updates may not occur. The lack of knowledge about oral health care among nursing staff is also supported by Wårdh et al. [33].

Nursing staff know that good nursing includes oral health care, but this knowledge does not always mean that oral health care is administered [2, 32]. In a study that was conducted by Wardh et al. [2] where 22 in-depth nursing interviews were administered, it was found that the quality of oral health care is largely dependent upon the cooperation of the elderly patients. Some nurses reacted in a negative way due to the risk of being bitten by elderly patients during oral health care. Patients who wore dentures were not always willing to take them out and cleaning dentures also seemed to be a repulsive activity. Some of the nursing staff in this study experienced a lack of time as a factor inhibiting good oral health care. Others did not see lack of time as a problem, but in stressful situations, oral health care could be easily forgotten. Ethical dilemmas can also become an issue. Some nursing staff worry about whether it is right or wrong to force oral health care when an elderly patient refuses care.

## 5. Implications for Nursing Care

Oral health care is an important part of treatment for all patients, particularly those who require assistance with activities of daily living. The majority of hospitalised patients within the acute medical units are older—over age 65 years [1]. Concerns regarding the nursing care older people receive in the acute care environment are frequently cited in the literature and in particular the link between patient outcomes and nursing care. In this paper we have focused on oral care; however other aspects of patient care require similar attention but are beyond the scope of this paper. As the population ages, the likelihood of altered physical ability and presence of disease increases, leading to a reduced ability to perform activities of daily living, for example, oral care. Comorbid conditions most likely to be seen in the older population include, but are not limited to, diabetes, congestive heart failure, renal disease, glaucoma, and cataracts. These isues can lead to the need for hospitalisation often resulting in a protracted length of stay and the increased chance of deconditioning which in turn prevents older patients from attending to basic care needs such as oral care. Hospitalization can represent the beginning of functional decline and increased dependency that may lead to an individual requiring long-term care [34–37]. The state of a patient's oral health can have a significant impact on their health outcomes, most notably psychosocial well-being, respiratory health, and nutritional status.

Oral care is often overlooked in the context of acute medical wards within hospitals. Routine oral care (tooth brushing, mouth toilets, etc.) are often the responsibility of the nurse or health assistant without the required knowledge and skill or comprehensive hospital protocols to follow. This responsibility is related to decreased functional decline leading to ability to attend to ADLs. The link between functional decline and the need to assist with ADLs may not always be apparent to the nurses caring for hospitalised older patients. To overcome this issue the literature on this subject has identified the need for nurses to routinely assess oral health status and to determine what assistance is required for the patient to maintain good oral health, especially for older patients as debility and frailty can interfere with a patient's ability to self-manage their oral care. 

Many patients are often admitted through the emergency department and as such may not come in with the basics—toothbrush and toothpaste. Nursing admission assessments do not routinely include assessment of the oral cavity or the patient's ability to manage self-care, and much of the assessment of oral care needs and self-care abilities of patients is subjective with decision support protocols not routinely available within the acute environment.

Anecdotal evidence suggests assessment of patient's oral health on medical in-patient wards is generally poor. Patient groups with specific needs often receive greater intervention with their oral care. Such interventions should be applied to all hospitalised patients. It is encouraging to note that the literature supports that when nurses are offered education and decision support they respond positively and actively engage [38–40]. 

## 6. Discussion/Conclusion

The available literature supports the view that oral health care of hospitalised patients is variable and overlooked and that nurses' knowledge and practice are variable. Nurses play a key role undertaking oral health care including the identification and evidence to guide the patients at risk for therapy-related oral mucositis [41], periodontal disease (a chronic inflammatory condition), chronic infection of the tissue surrounding the teeth, and assessment of patients' ability to independently manage their oral hygiene. Fundamental to this assessment are both an oral assessment and a thorough functional assessment. This requires the involvement of the nursing staff, especially in cases where oral care and any necessary dental treatment are vital to ensure medical treatment.

In the acute medical setting, oral health care seems to be separated from other nursing activities and is not discussed when nursing care plans are written, only when oral problems are obvious. To enhance the integration of oral care within routine nursing practice using a patient centred approach, some strategies may include education of staff, patient, and carers; provision and/or increased accessibility of equipment (toothbrush, toothpaste, and mouth wash); and inclusion of oral health care as a major component of all documentation of nursing care [2]. Policies and practices that support the maintenance of good oral health are needed to lessen the disease burden and promote healthful aging for this growing population [42]. Health care professionals need to reduce the obvious service fragmentation and collaborate, especially since the most severe oral problems are usually found in the older patients [1].
